# Editorial: Using virtual environments to understand behavior

**DOI:** 10.3389/fnbeh.2023.1156800

**Published:** 2023-03-24

**Authors:** Giuseppe Curcio, Giuseppe Placidi, Stefano Triberti

**Affiliations:** ^1^Department of Biotechnological and Applied Clinical Sciences, University of L'Aquila, L'Aquila, Italy; ^2^Department of Life, Health and Environmental Sciences (MESVA), University of L'Aquila, L'Aquila, Italy; ^3^Faculty of Human Sciences, Università Telematica Pegaso, Naples, Italy

**Keywords:** immersive technologies, Virtual Reality, behavioral neuroscience, psychological research, cognition

In the last decades, Virtual Reality (VR) has left highly specialized laboratories to become a common-use product. Immersive technologies have entered people's homes to support entertainment, education, and even healthcare, rehabilitation (Pizzoli et al., 2019; Riva and Serino, 2020) and psychological therapy, i.e., fear extinction (Dunsmoor et al., 2014; Maples-Keller et al., 2017). Many among the major players in innovation are turning to VR to re-think the technology-driven ways we socialize and work (Cerasa et al., 2022; Rospigliosi, 2022). However, VR does not cease to represent an invaluable tool that supports and inspires behavioral research.

The aim of this Research Topic has been to highlight the latest technologies, experimental techniques, and methods, involving virtual environments to investigate specific questions in behavioral neuroscience research.

The Research Topic features interesting research studies and proposes innovative ways to use virtual environments (VEs) within psychological research, highlighting that the design of mediated experiences has still a lot to offer to research methods.

Regarding the contributions in detail, Ben-Zeev et al. employed VR to modify two spatial learning tasks (Virtual Morris Water Maze-VMWM and Virtual Radial Arm Water Maze-VRAWM) and improved their ecological validity compared with the traditional, non-immersive, desktop-based counterparts. By means of the “Vizard 5 Virtual Reality” software both VMWM and VRAWM tasks were programmed. During the tasks, subjects had a first-person perspective of the room and could rotate the view as in head motion, thanks to the Oculus Rift DK2 VR device. A Microsoft X-Box controller to facilitate navigation in the environment and rotate the view completed the system. By using the implemented VR tasks, the Authors tested (and disconfirmed) the hypothesis that physical exercise would improve spatial learning in healthy participants.

Huynh et al. in their study demonstrated that Vicarious Trial-and-Error (VTE) improved during deliberation asked in a translational foraging task. The VR task was implemented on a computer by using the Unity 3D game engine featured on an Internet page. The authors showed the utility of VR tasks to identify multiple dimensions of decision-making across species. More specifically, results from the Movie Row and Candy Row tasks provided information on the differences between humans' and rodents' decision-making. When faced with difficult offers and when acting against one's own preferences, humans needed more time before deciding. They used longer pauses and enacted complex behavior (e.g., rotating, changing direction, or moving around). It can be concluded that during navigation in VR humans act a Vicarious Trial-and-Error behaviors, similarly to what has been seen in rodents during Restaurant Row: this could be seen as a behavioral correlate of deliberation process across species.

Arake et al. used an Immersive VE to implement a Dynamic Task Scenario for assessing task-related brain activity through ERPs. Participants moved through a VE with a first-person view and identified and reached the goal point by walking around the ruined outdoors and warehouses. Downward arrows floating in the air indicated the goal point: during the walking armed enemies hid behind obstacles, blocking the participant's view. The task was developed using a military training simulator (VBS3). Custom functions were implemented to synchronize the task with the EEG recording that was collected during the task with a 64-channel dry electrodes wireless EEG headset. Error related negativity (ERN) and correct- (response)-related negativity (CRN) elicited by shooting-related events were the dependent variables. Results showed that the ERN amplitudes correlated with individual shooting performance. The main brain source of such ERN was the rostral anterior cingulate cortex (ACC). These results could effectively contribute to the evaluation of cognitive functions and behavioral performance in a VE.

Tuena et al. have studied the egocentric and allocentric spatial memory in hallucinations due to Parkinson's disease (PD) by using VR in a case study. They used a VR navigation task with five different navigation conditions (passive, immersive, map, path decision, and attentive cues) by studying both a case study and a PD control group. The task was an immersive VR landmark-based navigation task composed of two phases: encoding and recall. The Authors found that, in general, the case study showed a dissociation between egocentric and allocentric performance being the former highly inadequate in both the “passive” and “attentive cues” conditions. Interestingly in the VR “immersive” condition, such dissociation disappeared and a trend toward a better performance for egocentric than allocentric memory emerged. These results suggest that body-based information evaluated trough VR navigation tasks could play a relevant role in PD hallucinations. Moreover, VR could help to understand the neurocognitive underpinnings of visual hallucinations, providing useful data about the mechanisms contributing to hallucinations and fundamental to design non-pharmacological interventions aimed at reducing hallucinations in PD patients.

Binder and Spoormaker used VR to quantify human avoidance behavior, a key symptom of most anxiety disorders. However, the quantification of avoidance behavior in human healthy participants is often performed by implementing avoidance tasks utilizing button responses or joystick navigation on the screen of a computer as indicators of such behavior. The Authors have developed a new automated realistic and immersive VR paradigm, in which participants can freely navigate in virtual 3-dimensional, 360-degrees scenes by real naturalistic body movements. By using VR, a differential fear conditioning procedure was possible to implement through three newly developed behavioral tasks in the following conditioned stimuli: an approach, a forced-choice, and a search task. The VE was developed in Unity 3D Pro with a sampling rate of 90 frames per second: VR was connected to Steam VR and was presented by using an HTC VIVE with controllers and headphones. Results demonstrated that the search task was sensitive to detect avoidance behavior after fear conditioning only, whereas the behavioral approach and forced-choice tasks were sensitive to more intense avoidance behavior after fear conditioning and additional reinforcement.

In conclusion, the papers published within the present Research Topic demonstrated that, through the immersion of participants in VEs, researchers could test behavioral responses, cognitive processes, and emotional states within complex scenarios that mirror real-life situations without sacrificing sophisticated monitoring and experimental control.

## Author contributions

All authors listed have made a substantial, direct, and intellectual contribution to the work and approved it for publication.
